# Supplementary material for: Dealing with mixed and non-normative traffic. An agent-based simulation with the GAMA platform
Source: PLoS One. 2023 Mar 6;18(3):e0281658. doi: 10.1371/journal.pone.0281658 (PMC9987808; doi:10.1371/journal.pone.0281658)
Supplement: Attachment — Submitted filename: response to reviewer - final.pdf [file pone.0281658.s003.pdf]

RESPONSE TO REVIEWERS

Reviewer #3: However, the authors need to pay careful attention to the following and revise the manuscript carefully before publishing can be done:

- Incomplete sentences e.g., line 333 page 10 "(Function :"

- Poorly formatted references in the text and poor latex formats e.g., line 300 page 10 "textbfchooseLane" and line 306 page 10 "see ??"

- Grammatical mistakes e.g line 117 page 4 e.g. having a bicycle or not

- Others

- Line 177 page 5, it is not clear if authors refer to the Table 1 in the article being referenced

- Need for consistency. Line 265 page 8. Compute_path written differently from other Java methods highlighted

These are just a few examples. Further proofreading is necessary for the whole manuscript.

Done.

Reviewer #3: Additionally, for the results it would be beneficial to readers if the authors include a brief explanation on the reason behind the amplified effects of oscillations of their model for motocycles in comparison to [45]. Especially since it happened only in the case of Tay Son street.

It is possible to observe more oscillations in our model than in that of [43] for motorcycles. One explanation is that the vehicles in the model of [43] are not based on lanes but on free spaces to move. Therefore, they have more possibility to execute maneuvers to avoid the vehicles stopped in front of them and therefore, if they decide not to stop at the red light (probability to stop at the red light of 0.5), to continue their way allowing less impact of the red light. In our model, the blocking of all lanes at the red light happens more quickly, leaving a more pronounced oscillation phenomenon.

We do not find this phenomenon for Chua Boc because the road is much less wide than Tay Son, so even if vehicles have more ability to avoid obstacles, the blocking of spaces at the level of red lights happens much faster and with the phenomenon of oscillation.
